# Establishment of a clinical workflow for in vivo Raman spectroscopy during head and neck cancer surgery

**DOI:** 10.1038/s41598-025-08222-9

**Published:** 2025-07-07

**Authors:** Ayman Bali, Thomas Bitter, Mussab Kouka, Jonas Ballmaier, Ines Latka, Florian Windirsch, David Pertzborn, Nadja Ziller, Marcela Mafra, Nikolaus Gaßler, Jürgen Popp, Anna Mühlig, Ferdinand von Eggeling, Orlando Guntinas-Lichius, Iwan W. Schie

**Affiliations:** 1 Department of Otorhinolaryngology, Jena University Hospital Department, Am Klinikum 1, 07747 Jena, Germany; 2 Leibniz Institute of Photonic Technology, Jena, Germany; 3 Section Pathology of the Institute of Forensic Medicine, Jena University Hospital, Jena, Germany; 4 Institute of Physical Chemistry and Abbe Centre of Photonics, Friedrich Schiller University Jena, Jena, Germany; 5 Department for Medical Engineering and Biotechnology, University of Applied Sciences Jena, Jena, Germany

**Keywords:** Cancer imaging, Head and neck cancer, Cancer, Optics and photonics, Biophotonics

## Abstract

As first part of an ongoing prospective feasibility trial (DRKS00028114) this work explored the integration of in vivo Raman spectroscopy (RS) into the routine setting workflow of head and neck cancer (HNC) surgery. In vivo RS was performed intraoperatively on 30 patients with HNC cell carcinoma and 10 patients with inflammatory diseases as a control group. A standardized process was established using a Raman system complied with stringent medical device regulatory standards. Spectra were collected in vivo from the tumor site, the tumor margins, and healthy tissue. The learning curve of the HNC team significantly improved measurement times from over 30 min initially to 2 min after 15 patients. Data from 35 patients were interpretable, demonstrating clear spectral differences between tumor and healthy tissues. The intraoperative in vivo RS workflow is now well established and is being used in the ongoing clinical trial.

## Introduction

**Fig. 1:**
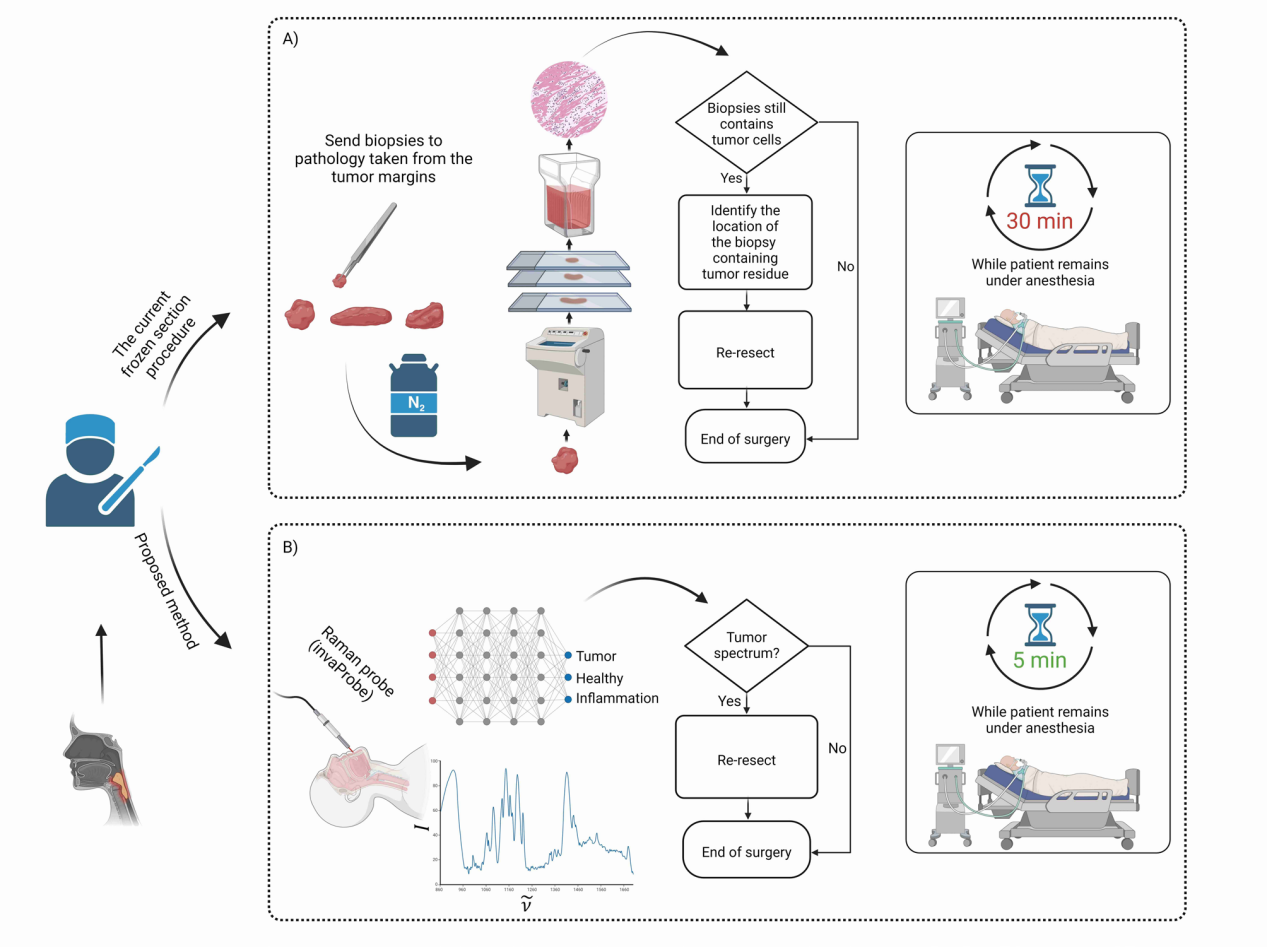
Comparison between the current frozen section procedure during tumor resection (**A**) and the proposed method using Raman spectroscopy (**B**). (**A**) The biopsies are snap frozen in nitrogen, sectioned, and stained. A pathologist examines the samples to determine if tumor cells are present. If tumor cells are detected, additional tissue is resected. The processing time for this method takes up to 30 min per probe while patient remains under anesthesia. (**B**) In the proposed future method using real-time Raman spectroscopy (Raman invaScope), no biopsies would be taken. Instead, measurements would be performed directly on areas suspected to contain tumor tissue. The spectra would be analyzed in real-time to detect the presence of tumor cells. If tumor spectra are identified, additional tissue would be resected. The processing time for this method would take up to 5 min while the patient remains under anesthesia. Created in BioRender. Ziller, N. (2025) https://BioRender.com/0blcdsw.

Head and neck squamous cell carcinoma (HNSCC) encompass a diverse group of malignancies. These cancers rank as the sixth leading cause of cancer-related mortality worldwide^1,2^. Treatment typically involves a comprehensive multidisciplinary strategy that includes surgery, radiation therapy, chemotherapy, immunotherapy, or a combination of these modalities. A common treatment approach is surgical removal, aimed at excising the tumor comprehensively^3,4^. Current methods for assessing tumor margins intraoperatively primarily involve histopathological techniques such as the frozen section analysis procedure (Fig. 1A). Although widely utilized, this method faces significant challenges, including limitations in accuracy (63.1–97.2%) and subjectivity, as well as lengthy processing times (approximately 20–30 min per probe) and a notable potential for sampling errors^5–8^. These issues not only increase the time and costs associated with surgery but also frequently lead to suboptimal surgical outcomes. This can manifest as either insufficient tumor margins or excessive removal of healthy tissue, ultimately affecting patient recovery and prognosis^8–11^.

In response to these challenges, recent advancements in photonic technologies have introduced new possibilities for enhancing surgical precision. Among these, Raman spectroscopy has emerged as a particularly promising tool due to its ability to provide real-time, label-free molecular insights into tissues^12^. Raman spectroscopy captures a unique molecular ’fingerprint’ of tissues, enabling the differentiation of malignant from non-malignant tissues almost instantaneously^13–23^ with high sensitivity (85–92%) and specificity (78–89%)^24^. Raman spectroscopy has been applied both in vivo and ex vivo across various organs, including the esophagus, stomach, colon, cervix, lung, brain, nasopharynx, larynx, and the oral cavity where it has been instrumental in detecting precancerous and cancerous changes^25^. The non-destructive, label-free, and non-ionizing nature of the technique keeps the specimen safe, while the rapid analysis capabilities make it especially suitable for integration into surgical workflows, offering the potential to significantly reduce the duration of surgery and improve surgical accuracy^26–40^.

HNSCC has been a significant application area for Raman spectroscopy. Both i*n vivo* and ex vivo Raman spectroscopy have demonstrated the ability to distinctly differentiate between normal tissue, oral dysplasia, the spectrum of oral potentially malignant disorders, and tumor tissues. This differentiation is achieved through the identification of specific changes in the spectroscopic signature related to common macromolecules, such as molecular bands linked to lipid and protein vibrations, which results in high diagnostic sensitivity and specificity^41–55^. However, it is important to note that the cited studies primarily involve laboratory-based research, which, while instrumental, is still in the process of being integrated into routine clinical practice. Moreover, existing in vivo studies, if available, typically cover only specific parts of the head and neck area, rather than encompassing the entire region.

Furthermore, none of these studies involve systems which adhere to the requirements set out by the Medical Device Regulation MDR2017/745 or bearing a CE mark, highlighting a significant gap in the transition to clinical application.

Our current RAMAN-HNSCC clinical trial differs significantly in its approach (Fig. 1B) and focus from previous applications, because it is particularly aiming to integrate Raman spectroscopy into surgical workflows for head and neck cancer surgeries. This trial seeks not only to validate the efficacy of Raman spectroscopy as a supportive diagnostic tool during operations but also to refine the precision of tumor margin determinations—a critical factor in surgeries aimed at balancing the complete excision of tumors with the preservation of healthy tissue to minimize morbidity and functional or aesthetic compromise. As a first step, it was necessary to establish a workflow for the Raman measurements optimally fitting into the process of head and neck cancer surgery.

In summary, while previous research has established a solid foundation for the clinical application of Raman spectroscopy, our study aims to extend its utility by enhancing real-time analysis techniques for intraoperative use. We conducted a comprehensive analysis across various tumor sites in the head and neck region, incorporating control groups with inflammatory changes to further refine diagnostic accuracy. Our work aimed to assess the integration of Raman spectroscopy into surgical procedures, specifically evaluating its feasibility and efficiency within the stringent time limits of operative settings. A key strength of our study is the use of a device which adhere to the requirements set out by the Medical Device Regulation MDR2017/745 and approved for this clinical trial by the Federal Institute for Drugs and Medical Devices (Bundesinstitut für Arzneimittel und Medizinprodukte, BfArM), ensuring the reliability and safety of the technology.

## Materials and methods

### Study design

The European medical device regulations (MDR EU 2017/745) and the national Medizinprodukterecht-Durchführungsgesetz [MPDG] regulate the translation of non-CE certified medical devices for investigative clinical studies, including for fundamental research questions for purely scientific reasons. The requirements regarding other clinical investigations are outline in Article 82 of the MDR, with a reference to the defined regulation for other clinical investigations, which are outlined in the MPDG. To requirements must be followed, i.e. a positive opinion has been issued by the competent ethics committee in accordance with section 52(1) MPDG; and the other clinical investigation has been notified to the competent federal higher authority in accordance with section 53(1) MPDG. Therefore, such an investigation has to be authorized by the responsible federal higher authority and the responsible ethics committee. The higher authority in Germany is the Federal Institute for Drugs and Medical Devices (Bundesinstitut für Arzneimittel und Medizinprodukte [BfArM]). The BfArM approved the study (DE-22-00014291). The RAMAN-HNSCC study has also been approved by the ethics committee of Jena University Hospital (Registration No.: 2022-2758-MDPG_sonst_mono) and is registered as a clinical study in the German Clinical Trials Register under the identifier DRKS00028114 (first trial registration: 10/11/2022). All methods were performed in accordance with the relevant guidelines and regulations. The study is ongoing and designed as a prospective, monocentric study. It primarily aims to assess the feasibility and safety of using the in house build Raman device (Raman invaScope) for intraoperative spectroscopic examinations of HNSCC. The spectroscopic data obtained intraoperatively will be compared with postoperative ex vivo analyses to validate the device’s diagnostic capabilities. Although the primary aim of the study is to show the feasibility, a power analysis was performed to allow a better planning of the subsequent clinical trials. To allow to detect at least one Raman peak to differentiate between non-tumorous and tumor tissue with an Cohen’s d effect size of 0.5, and a ratio of tumor patients to volunteers of 1.5:1, with a power of alpha = 0.7 and significance level of *p* = 0.05 for two-sided testing, a final sample of at least 50 patients and 30 healthy controls (intention-to-treat) will be needed.

### Recruitment and follow-up

This ongoing monocentric study is being conducted at the Department of Otorhinolaryngology, Jena University Hospital, Jena, Germany. The primary objective is to evaluate up to 100 patients with HNSCC. Additionally, for control purposes, 40 patients with inflammatory conditions in the oral cavity, oropharynx, hypopharynx, or larynx will be screened. To date, 30 HSNCC patients and 10 patients with uncharacteristic inflammatory conditions (control group) have been enrolled.

For each patient, a final histopathological assessment (Table 1) was performed to confirm diagnoses. Squamous cell carcinoma (SCC) was the most prevalent diagnosis, identified in 25 patients. The biopsies from the remaining five patients showed no signs of cancer and these patients were subsequently excluded from the study.

**Table 1:** Comprehensive overview of patient distribution and diagnostic characteristics across different tumor locations in head and neck cancer, including subgroup specifications, 8th edition TNM classification, and histopathological outcomes as a gold standard diagnostic statement. SCC = squamous cell carcinoma.

No	Group	Subgroup	TNM	Histopathology
1	Oral cavity	Tongue	T2 N2c MX	SCC
2	Oropharynx	Tonsil	T4a N1 M0	SCC
3	Oropharynx	Tonsil	T2 N1 MX	SCC
4	Larynx	Glottis	T1 N1 M0	SCC
5	Oropharynx	Tonsil	T4a N2b MX	SCC
6	Larynx	Glottis	T3 N2 M0	SCC
7	Hypopharynx	Sinus piriformis	T3 N0 M0	SCC
8	Oropharynx	Tonsil	T2 N0 M0	SCC
9	Oral cavity	Tongue	T2 N2b M0	SCC
10	Oropharynx	Tonsil	T3c N2c M0	SCC
11	Hypopharynx	Sinus piriformis	T2N N2b M0	SCC
12	Oropharynx	Tonsil	T2 N0 M0	SCC
13	Hypopharynx	Sinus piriformis	T4 N3b M1	SCC
14	Oropharynx	Tonsil	T4 N2 M0	SCC
15	Larynx	Supraglottis	T4 N2 M0	SCC
16	Oropharynx	Tonsil	T2 N1 M0	SCC
17	Larynx	Glottis	T1 N0 M1	SCC
18	Oral cavity	Tongue	T3 N2 MX	SCC
19	Larynx	Glottis	T2 N0 M0	SCC
20	Oral cavity	Tongue	T3 N3b M0	SCC
21	Larynx	Glottis	T1 N0 MX	SCC
22	Oropharynx	Soft palate	T1 N1 M1	SCC
23	Oral cavity	Tongue	T1 NX M0	SCC
24	Larynx	Glottis	T1 N0 MX	SCC
25	Oral cavity	Tongue	T3 N2 MX	SCC

### Raman invaScope design and surgery

**Fig. 2:**
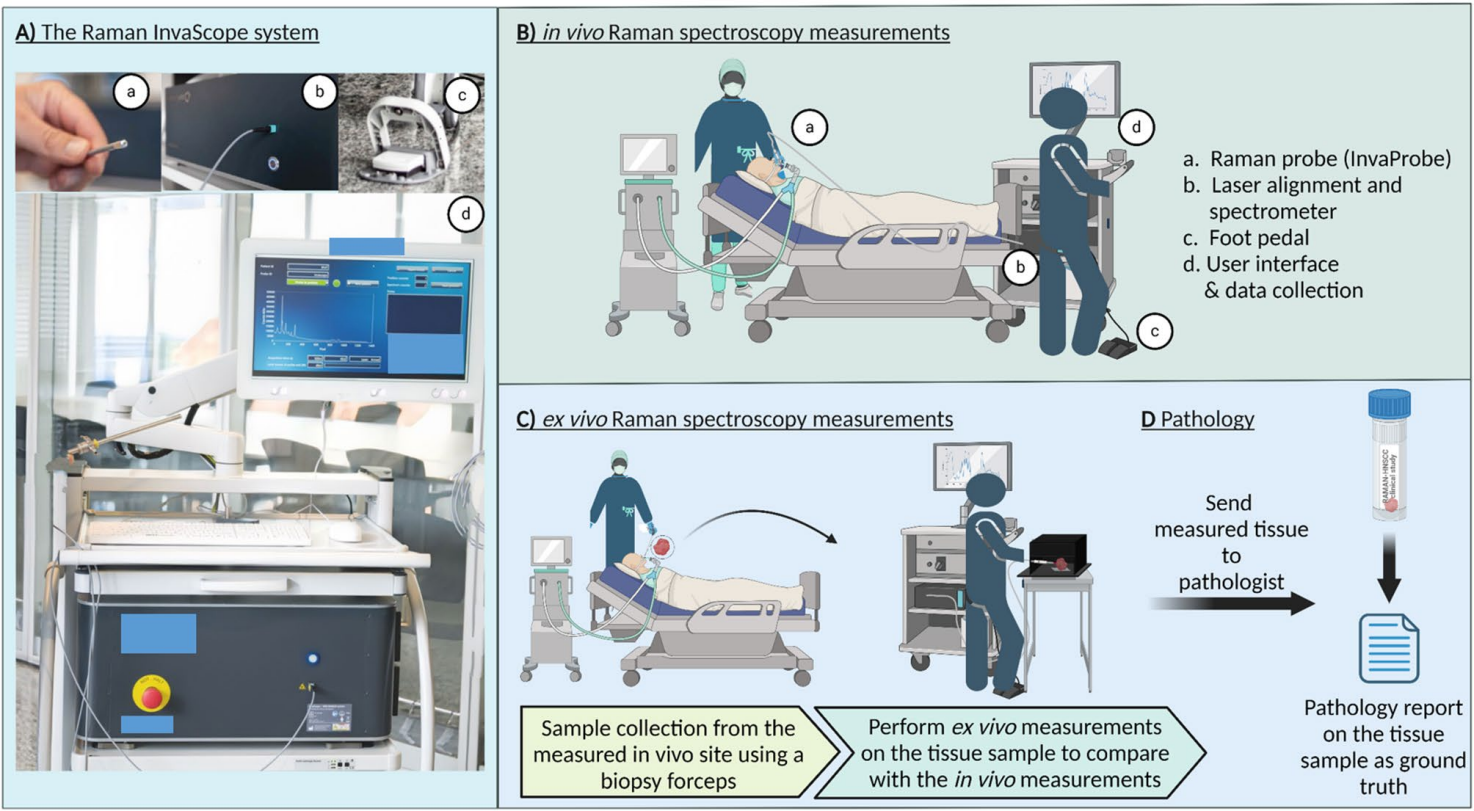
(**A**) The Raman invaScope system. (**B**) In vivo Raman spectroscopy measurements is performed, using the fiber-optic probe (a), which is connected to the invaScope system that is equipped with a narrowband laser for excitation and a spectrometer for the data acquisition (b). The acquisition is started by pressing the foot pedal (c) and the data is displayed on the screen (d). (**C**) Sample collection and ex vivo Raman spectroscopy measurements on the biopsies of the measured sites. (**D**) Spectroscopically measured tissue samples are sent to pathology for histopathological validation. Created in BioRender. Ziller, N. (2025) https://BioRender.com/pmbzye3.

The Raman invaScope (Fig. 2A) developed by the Multimodal Instrumentation Group at the Leibniz Institute for Photonic Technologies e.V. (Leibniz-IPHT) in Jena, Germany, is a Raman spectroscopy platform designed for in vivo clinical diagnostics. For practical deployment, the system was installed on a medical cart, facilitating mobility and flexibility in various clinical environments such as operating rooms and outpatient clinics. It is important to note that the Raman invaScope was configured for point measurements of tissue pathologies and does not support imaging due to the lengthy process required to generate hyperspectral Raman images, attributable to the low Raman scattering cross sections^25^. The system is based on an in-house developed system, which consists of a medical graded cart (Compact-cart Basis “Profi” with isolating transformer, iTD GmbH), a medical graded computer (Medical Line THA.leia^3^ 21.5” Touch, MCD Medical Computers Deutschland GmbH), which runs the software, which controls the device and the base-unit of the invaScope device. The base-unit primarily hosts a narrrow-band excitation laser with a wavelength of 785 nm (FER-785, Princeton Instruments), a CCD camera (Pixis 100, Princeton Instruments) and an imaging spectrograph (Acton Series LS 785, Princeton Instruments). Furthermore, there are multiple components integrated, which ensure a proper assessment of the intensity of the excitation laser emission, as well as micro-controller, to ensure proper coordination between the components. The device operates within a wavenumber range of 500 cm^−1^ to 3300 cm^−1^, and a spectral resolution of approximately 20 cm^−1^. The fiber optical probe is designed in a so-called 10 around 1 design, meaning that at the center is the excitation fiber surrounded by 10 fibers, which are used for the collection of the generated Raman signal. As it is a non-CE certified device designed for a clinical investigations, a variety of test according to relevant norms were performed in certified test houses. It has passed EMC test (EN 60601-1-2), Electrical safety (EN 60601-1) and Laser safety (EN 60601-1-22).

The user interface of the Raman invaScope was designed to be intuitive, allowing users without technical expertise to operate the system effectively. Furthermore, an initial training was performed, to ensure correct and most efficient used of the system. The software provided a step-by-step guide from the calibration of the Raman probe to the initiation of measurements. Compliance with the Medical Device Regulation (MDR 745/2017) and relevant standards was ensured, permitting the acquisition of Raman spectra directly from patient tissues. The probe component of the system was designed for multiple sterilizations, supporting reuse in a clinical setting.

### Data collection and analysis

Raman data pre-processing and analysis were conducted using a custom-developed Python script specifically designed to optimize spectral data and enhanced analytical accuracy. Biological samples, particularly tissues, contain significant background contributions from tissue autofluorescence and Rayleigh scattering, which can overshadow the low-intensity Raman signal. To address this, the spectra were first corrected for dark current and the constant offset bias of the CCD detector using recorded dark spectra. Additionally, the spectra were corrected for cosmic spikes using an in-house developed algorithm that combines differential peak detection and interpolation between adjacent pixel values. The resulting spectra were intensity-corrected based on measurements from an intensity calibration standard, specifically a National Institute of Standards and Technology (NIST)-traceable calibration standard SRM-2241 for Raman spectroscopy for an excitation wavelength of 785 nm. Following calibration, background correction was performed using an asymmetric least squares (ALS) approach^56^. Given that the acquired spectra included measurements with either uncorrectable background or no Raman signal, the data was further cleaned using cross-correlation between the dataset’s mean spectrum and each individual spectrum. The corrected spectra were then area-normalized and presented as mean spectra with standard deviation for the individually selected groups.

### Measurement process

The invaScope system is initialized before bringing the patient to the surgery room to prevent delays in the surgical room plan. Measurements start once the patient is under general anesthesia. Initially, in vivo Raman spectroscopy measurements are taken at various sites including tumor or inflammation areas, healthy tissues, and transitional zones (Fig. 2B). Following this, tissue samples from these locations are collected using biopsy forceps. These samples are then measured ex vivo in a black box to remove interference and validate the in vivo data (Fig. 2C). Subsequently, the samples are sent to the pathology department (Fig. 2D) where a detailed assessment is conducted, providing a pathology report that serves as the ground truth. This report is used to evaluate the accuracy and diagnostic utility of the Raman spectroscopy results.

## Results

### Intraoperative Raman measurements

The in vivo Raman spectroscopy measurements were performed directly during surgery. Tissue samples were then collected from the same sites where measurements were taken, using a biopsy forceps. These tissue samples were subsequently sent in formalin to the pathology for routine tissue processing and detailed histopathological examination, which serves as the gold standard for verifying the characteristics of the measured site. After collection, additional ex vivo measurements were performed on these samples to compare with the in vivo data.

**Fig. 3:**
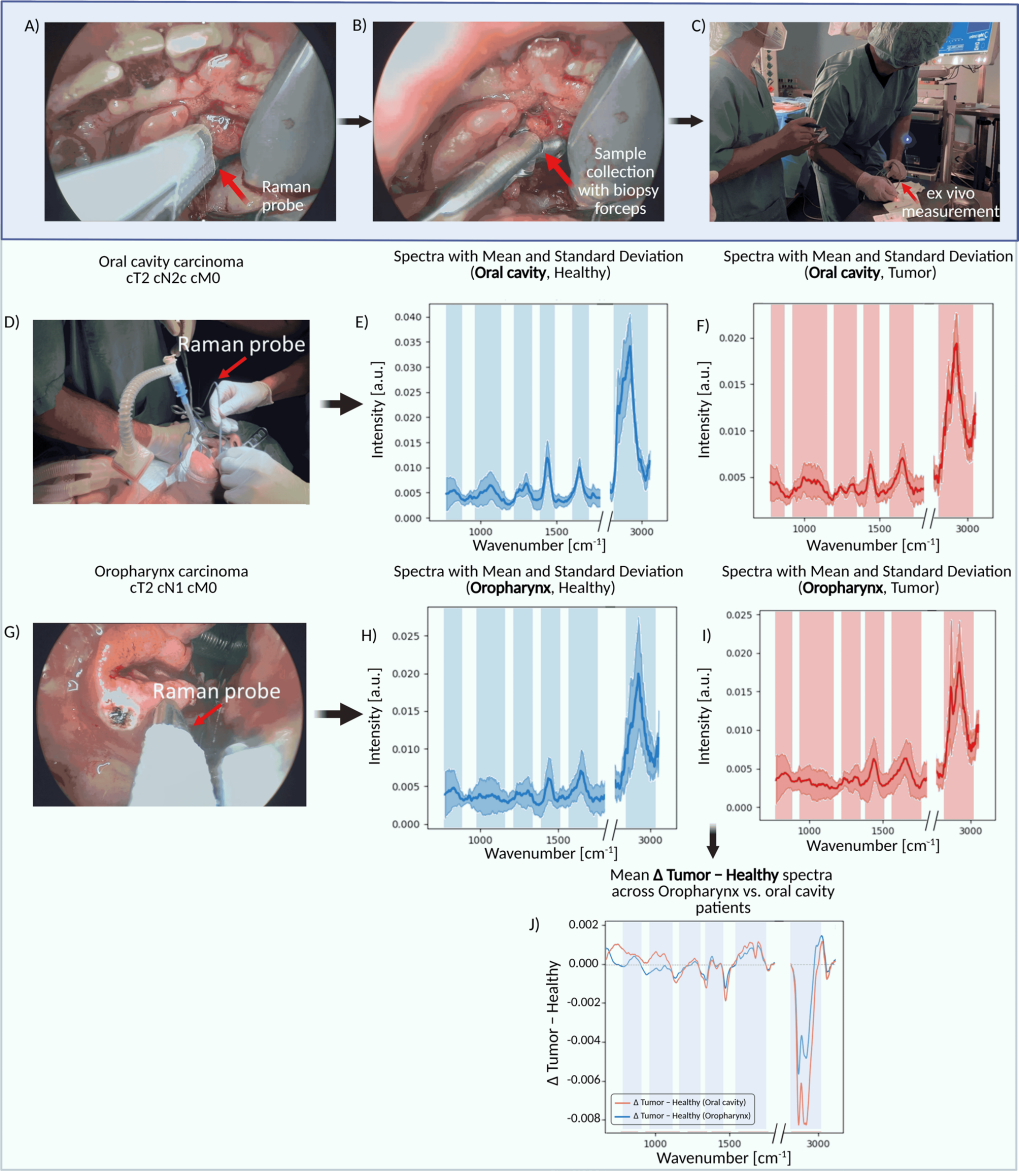
Above: Illustrating the measurement process of Raman spectroscopy during surgical procedures. (**A**) Positioning of the Raman probe (Red arrow) on the tumor site at the anterior medial floor of the mouth for measurement. (**B**) Sample collection from the measured in vivo site using a biopsy forceps (Red arrow) to send for histopathological evaluation. (**C**) Applying ex vivo measurements on the collected tissue samples to compare with the in vivo measurements, the ex vivo measured tissue samples were then sent to pathology department for histopathological examination. The carcinoma of the floor of the mouth was classified as T4a cN1 cM0. Below: Raman measurements from two different locations with (**D**) Oral cavity carcinoma, (**G**) Oropharynx carcinoma. In the right section: Mean Raman spectra with single standard deviation from 10 patients with oral cavity carcinoma and 10 patients with oropharynx carcinoma: (**E**) healthy tissue in the oral cavity, (**F**) tumor tissue in the oral cavity, (**H**) healthy tissue in the oropharynx, (**I**) tumor tissue in the oropharynx, respectively. Marked areas in Figure (**E**–**I**) highlight the peak regions which contain the biological information. (**J**) Mean difference spectrum (tumor minus healthy) averaged across all so far included patients in the oropharynx and oral cavity cohorts, with the most pronounced spectral contrasts indicated in both the fingerprint and high-wavenumber regions. Created in BioRender. Ziller, N. (2025) https://BioRender.com/hw7vge1.

The three aspects, i.e. the in vivo measurements with the Raman probe, the collection of tissue samples from the measured sites and the ex vivo measurements on the collected tissue samples are shown in Fig. 3A–C for a cancer of the floor of the mouth.

Raman spectroscopy was applied to various anatomical sites within the oral cavity, oropharynx, larynx, and hypopharynx to assess its utility in distinguishing between tumor and healthy tissues. Spectra were collected from the tumor site, the tumor margins, and healthy tissue. For each measurement site, between 8 and 15 spectra were collected with each spectrum being background corrected and denoised. Figure 3D,G serves as a visual representation of the flexibility of the Raman invaScope during use in surgical settings. The spectra displayed in Fig. 3E–I are derived from measurements conducted on 10 patients with oral cavity carcinoma and 10 patients with oropharynx carcinoma. The data is shown with both the mean values and single standard deviations to illustrate the reproducibility and consistency of the measurements. This demonstrates the ability of the probe to generate reliable, distinct spectral profiles for both tumor and healthy tissue in the oral cavity and oropharynx. Marked areas in Fig. 3E–I highlight the peak regions which contain the biological information. Figure 3J displays the difference spectra (tumor − healthy) averaged across all so far included patients in the oropharynx and oral cavity cohorts, highlighting the most pronounced spectral contrasts in both the fingerprint and high-wavenumber regions. Detailed band assignments are provided in the Discussion.

### Workflow, feasibility and learning curve

**Fig. 4:**
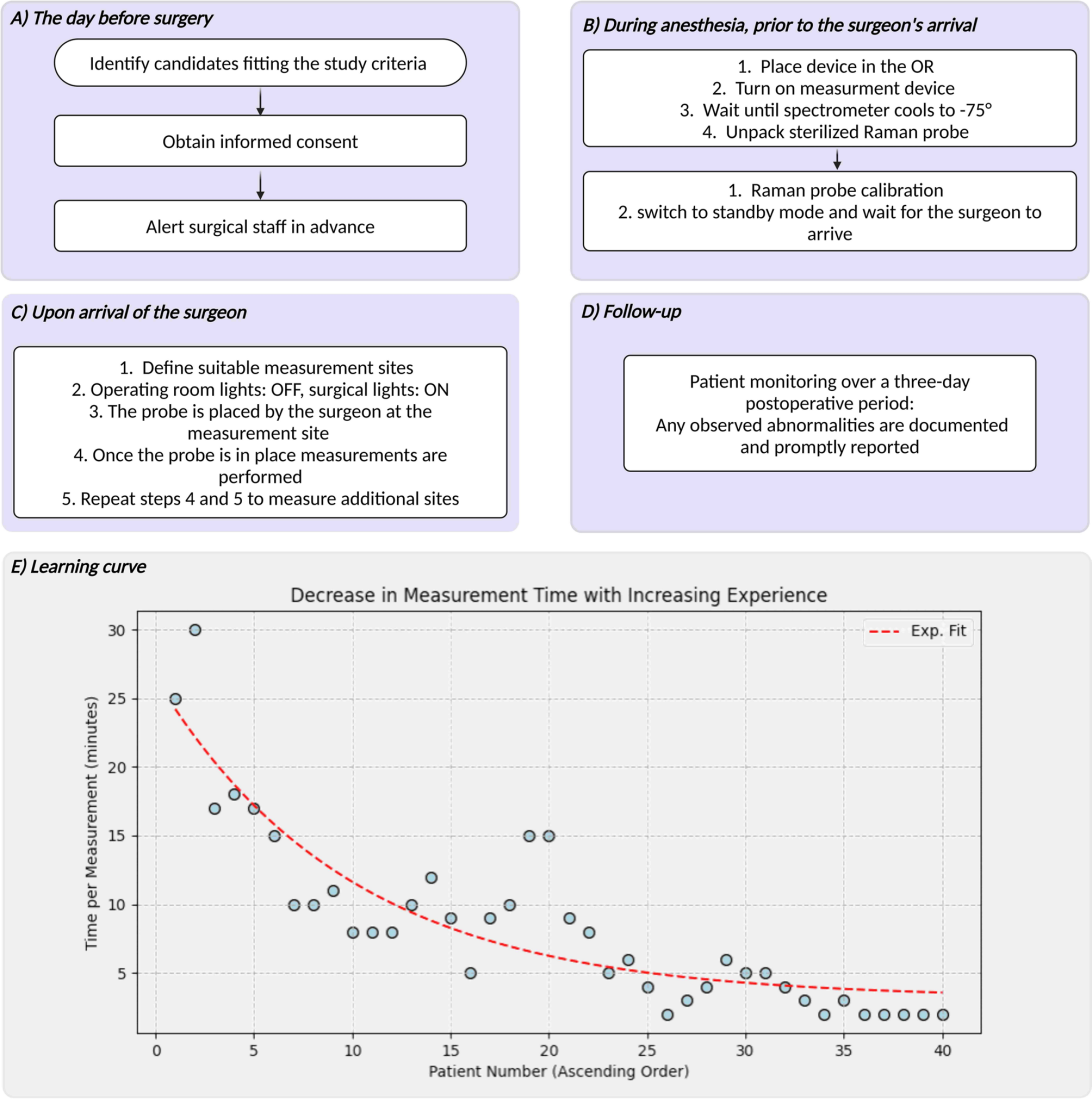
(**A**–**D**) Established workflow diagram for integrating Raman spectroscopy in surgical procedures, (**A**) The day before surgery, (**B**) During anesthesia, prior to the surgeon’s arrival, (**C**) Upon arrival of the surgeon, (**D**) Follow-up. (**E**) Learning curve of Raman spectroscopy measurement time over number of measured patients in a clinical setting during surgery. Created in BioRender. Ziller, N. (2025) https://BioRender.com/qoyqr88.

We optimized our initial concept during implementation to integrate the Raman invaScope into intraoperative head and neck surgery, as shown in Fig. 4A–D. This workflow allows reproducible, efficient measurements without disrupting surgery. We evaluated the learning curve for surgical teams adopting the Raman invaScope. Initially, integrating this technology was challenging, but as the staff became more familiar with Raman spectroscopy, their proficiency improved. This significantly reduced measurement times, as depicted in Fig. 4E. Initially, measurements took over 30 min but were reduced to under 5 min after 15 patients, with current operations achieving approximately 2 min. This reduction demonstrates the surgical team’s adaptability and suggests these efficient measurement times will become standard. In the three days post-surgery, 8 adverse events were reported, unrelated to Raman measurements and typical of cancer surgery.

## Discussion

Our study demonstrated that integrating Raman spectroscopy into the surgical workflow requires meticulous technical preparation, clear interdisciplinary coordination, and several workflow innovations (cf. Figure 4A–D). During intraoperative measurements, ambient illumination poses a significant challenge. These light sources can create spectral interference, resulting in spectral artifacts that obscure the weak Raman signal and reduce the predictive accuracy of the analysis^25^. Additional challenges include the need to switch off imaging lights during Raman measurements, which increases the risk of tissue puncture and complicates the visual confirmation of spectral acquisition points^57,58^. To overcome these issues, we mounted the Raman probe onto an endoscopic white-light camera, allowing continuous visual guidance; once the probe was positioned at the target site, only the camera light was transiently extinguished for spectral acquisition. The spectrometer requires up to 10 min to cool its detector to − 65 °C, so we powered on and calibrated the device during anesthesia induction to ensure it was ready when the surgeon arrived. Finally, the operating team underwent hands-on training—conducted during the first patient cases—to distinguish high-quality Raman spectra from motion and ambient-light artifacts. Together, these measures enabled seamless integration of Raman measurements without compromising surgical flow or patient safety.

Concerning the learning curve (cf. Figure 4E), initial measurement times of up to 30 min per patient reflected the need to trial different light settings and probe-placement techniques across varied anatomical sites (e.g., oral cavity vs. hypopharynx), and to translate spectroscopist terminology (“acceptable signal-to-noise ratio,” “background artifact”) into practical guidance for surgeons. After approximately 10–15 cases, the team had optimized key parameters—such as exposure time, contact force, and camera-guided alignment—and streamlined the device cooldown and calibration process. As a result, average measurement times dropped below 10 min by patient no. 15 and under 5 min by patient no. 30. Based on these insights, future surgical teams aiming to integrate Raman spectroscopy may consider organizing brief pre-clinical simulation workshops to practice probe handling and spectrum assessment, developing concise checklists for setup parameters and spectrum-quality criteria, and embedding spectrometer startup (cooling and calibration) into routine anesthesia and surgical checklists. This rapid proficiency gain underscores the feasibility of translating Raman spectroscopy into routine clinical practice.

While previous studies focused on specific anatomical sites within the head and neck area^59–63^, our study includes measurements across multiple regions—namely the oral cavity, oropharynx, larynx, and hypopharynx. By examining a wider array of tissue types and anatomical complexities, our approach enhances the generalizability of our findings and provides a robust validation of Raman spectroscopy’s utility across the full spectrum of head and neck oncological conditions. A detailed breakdown of patient characteristics is presented in Table 1. This table categorizes patients into groups based on the tumor location and specifies the precise site within these locations where measurements were conducted. Additionally, the TNM classification provided in the table offers insights into the tumor size, presence of metastasis, and lymph node involvement. The histopathology column presents the pathology reports from the measurement sites, serving as diagnostic statements. This table serves as evidence of the versatility of the invaScope Raman system and probe, demonstrating the capability to measure diverse regions within the head and neck area effectively.

Importantly, our device is in accordance with requirements for clinical investigational devices outline in the Medical Device Regulation (MDR) 745/2017, ensuring that our methodology meets the necessary standards of quality and safety in medical device use. It should be noted that, to date, there is no approved device specifically designed for this application within the head and neck regions. This highlights the relevance of our study and the potential for our compliant Raman spectroscopy device to address a significant need in clinical diagnostics. By adhering to EU MDR standards, we emphasize the reliability and clinical applicability of our findings, supporting the potential for future integration of Raman spectroscopy in routine oncological practice.

Moreover, we have included a selection of Raman spectra from our study to demonstrate the spectral characteristics encountered under different conditions. So far we can only report on individual cases. As far as the planned patient recruitment has been finished, the detailed data analysis will be performed and published. The present study was an important milestone for standardization of the in vivo Raman analysis. In our preliminary investigations within the fingerprint region (800–1800 cm^−1^), we noted distinct differences between healthy and neoplastic tissues (Fig. 3). The spectral characteristics observed closely align with findings reported by Cals et al. regarding oral cavity squamous cell carcinoma and healthy tongue tissue. Notably, specific peaks at 1003, 1032, 1266, 1448, and 1656 cm^−1^ appear in our spectra with deviation up to ± 15 cm^−1^. This deviation is below the spectral resolution of the system and is therefore not significant^41^.

In addition to these findings in the fingerprint region, we also noted differences between healthy and tumor tissues in the high wavenumber region (2800 to 3000 cm^−1^). Our study employed also comparative analysis with the spectral characteristics previously reported by additional literature^19,22,64–74^. The band located at around 1003 cm^−1^ is primarily related to Phenylalanine (protein), while the band observed at 1084 cm^−1^ is associated with a phosphate stretching mode and common in lipids or nucleic acids. The peaks at 1260 cm^−1^ is attributed to lipids, protein and amide III (in-plane deformation). Furthermore, the peaks at the region between 1570 and 1665 cm^−1^, related to *α*-helix of amide I, is associated with bending and scissoring motions of proteins, as well as the ring-breathing modes of DNA and RNA. Furthermore, the 1665 cm^−1^ band is primarily related to C=C vibration and therefore is associated with unsaturated fatty acids and can be used in combination with the 1444 cm^−1^ band to estimate the average degree of fatty acid unsaturation. Additionally, the peak observed at 1335 cm^−1^ corresponds to the deformation of DNA and RNA (Guanine specifically). Meanwhile, the peak at 1730 cm^−1^ is attributed to the C=O vibration, predominantly found in esters, phospholipids, and esterified fatty acids. The peaks at 2865 cm^−1^ corresponding CH_2_ stretching vibrations, which are primarily due of esterified lipids and fatty acids, and to a lesser degree to proteins. Proteins are more directly characterized by the 2920 cm^−1^, which is assigned to the CH_3_ stretching vibration and is highly representative of proteins.

Our comparisons serve as a validation point, confirming that the spectra indeed capture biological information relevant to the examined sites. This correlation with literature validates the effectiveness of Raman spectroscopy in conducting detailed molecular analysis and provides comprehensive insights into the structural and functional dynamics of tissue and cells.

Our study has enrolled 30 tumor patients in the investigative group and 10 patients in the control group with inflammation. Over three days post-surgery, 8 adverse events were reported but were typical for cancer surgeries and unrelated to the fiber probe-based Raman measurements. As we finish recruiting, we’re preparing a paper on a classification model based on the spectral data collected. While promising, the small sample size and the need for further research into long-term impacts of this technology on patient care are limitations of our study.

A number of challenges still need to be overcome before Raman spectroscopy can be integrated into everyday intraoperative practice. Since Raman spectroscopy is a point-based rather than a whole-field imaging modality, it takes time to measure several spots or when measuring repetitively. Most important, it does not provide comprehensive margin assessment if not applied in combination with other technologies. A potential solution could be the integration Raman spectroscopy with a whole-field imaging technique such as hyperspectral imaging or intraoperative fluorescence imaging using tumor-specific targeting agents^75,76^. These whole-field imaging techniques provide broad coverage but may yield false-positive signals. A combination with Raman spectroscopy could allow for improved specificity while maintaining the benefit of large-area visualization. Furthermore, specimen-based analysis of resection margins is recommended to ensure not only tumor-negative margins but also a > 5 mm tumor-free margin^77^. If Raman spectroscopy is used only for margins around the resection plane and for wound bed analysis, it cannot provide information on whether a sufficient tumor-free margin is present on the excised specimen. This is critical because a negative wound bed does not necessarily indicate a safe tumor-free margin on the resected tissue. A combined approach incorporating specimen-based analysis (performed by the pathologists) will further remain the gold standard, with Raman spectroscopy (may be in combination with a wide-field technology applied by the surgeon) serving as a complementary tool. Finally, real-world implementation challenges, including cost, and regulatory hurdles have to must be overcome. The EU MDR conformity of the study will facilitate the transfer to a clinical application, but a (start-up) company is needed to take over the process of product approval as a medical device. Then, it has to be shown that a use of the device has a clinical impact, hence to reduce positive margins and at best to improve overall survival. This will take a further series of clinical trial and a long way to go. This is the prerequisite for applying for reimbursement from the health insurance companies in different countries when using the technology.

## Conclusion

The present study has effectively demonstrated the integration of in vivo Raman spectroscopy into the surgical workflows for head and neck squamous cell carcinoma. The technology can be seamlessly integrated into existing surgical protocols, offering real-time, accurate molecular insights without disrupting operational flow. Compliant with strict medical regulatory standards, the system effectively addresses a range of challenges that typically complicate the translation of new medical technologies, such as Raman spectroscopy, to in vivo surgical studies. This ensures rapid data acquisition and analysis, avoiding any extension of surgery durations. Our workflow innovations have been crucial in adapting this new technology to clinical settings. These innovations ensure a uniform workflow that not only makes the measurements reproducible but also guarantees that the captured signals reflect biological information without interference from background signals that could disrupt the measurement. In the next phase, our focus will shift toward a detailed analysis of these spectral differences. The upcoming detailed classification model will be crucial for further validating the effectiveness of Raman spectroscopy in clinical applications and could lead to substantial improvements in clinical outcomes.

## Data Availability

The original contributions presented in the study are included in the article. Further inquiries can be directed to the corresponding author.

## References

[1] Holmstrup, P., Vedtofte, P., Reibel, J. & Stoltze, K. Long-term treatment outcome of oral premalignant lesions. *Oral Oncol.* **42**(5), 461–474 (2006). 16316774 10.1016/j.oraloncology.2005.08.011

[2] Myers, J. & Hanna, E. *Cancer of the Head and Neck* (Lippincott Williams & Wilkins, 2016).

[3] Hamman, J. et al. Impact of close margins in head and neck mucosal squamous cell carcinoma: A systematic review. *Laryngoscope* **132**(2), 307–321 (2022). 34143492 10.1002/lary.29690

[4] Rogers, S. N. et al. Survival following primary surgery for oral cancer. *Oral Oncol.* **45**(3), 201–211 (2009). 18674959 10.1016/j.oraloncology.2008.05.008

[5] Smits, R. W. H. et al. Intraoperative assessment of the resection specimen facilitates achievement of adequate margins in oral carcinoma. *Front. Oncol.* **10**, 614593 (2020). 33425769 10.3389/fonc.2020.614593 PMC7786304

[6] Gandour-Edwards, R. F., Donald, P. J. & Wiese, D. A. Accuracy of intraoperative frozen section diagnosis in head and neck surgery: Experience at a university medical center. *Head Neck* **15**(1), 33–38 (1993). 8416854 10.1002/hed.2880150108

[7] Long, S. M. et al. Use of intraoperative frozen section to assess final tumor margin status in patients undergoing surgery for oral cavity squamous cell carcinoma. *JAMA Otolaryngol. Head Neck Surg.* **148**(10), 911–917 (2022). 35925571 10.1001/jamaoto.2022.2131 PMC9353701

[8] DiNardo, L. J., Lin, J., Karageorge, L. S. & Powers, C. N. Accuracy, utility, and cost of frozen section margins in head and neck cancer surgery. *Laryngoscope* **110**(10 Pt 1), 1773–1776 (2000). 11037842 10.1097/00005537-200010000-00039

[9] Layfield, E. M., Schmidt, R. L., Esebua, M. & Layfield, L. J. Frozen section evaluation of margin status in primary squamous cell carcinomas of the head and neck: A correlation study of frozen section and final diagnoses. *Head Neck Pathol.* **12**(2), 175–180 (2018). 28836224 10.1007/s12105-017-0846-6 PMC5953870

[10] Serinelli, S., Bryant, S. M., Williams, M. P. A., Marzouk, M. & Zaccarini, D. J. Frozen-permanent section discrepancy rate in oral cavity and oropharyngeal squamous cell carcinoma. *Head Neck Pathol.* **16**(2), 466–475 (2022). 34655410 10.1007/s12105-021-01385-7 PMC9187809

[11] Sivrice, M. E. et al. Frozen section evaluation for surgical margins in laryngeal squamous cell carcinoma: Is it a reliable method for partial and total laryngectomies?. *Head Neck Pathol.* **17**(1), 172–177 (2023). 36171534 10.1007/s12105-022-01485-y PMC10063756

[12] Krafft, C., Schie, I. W., Meyer, T., Schmitt, M. & Popp, J. Developments in spontaneous and coherent Raman scattering microscopic imaging for biomedical applications. *Chem. Soc. Rev.* **45**(7), 1819–1849 (2016). 26497570 10.1039/c5cs00564g

[13] Austin, L. A., Osseiran, S. & Evans, C. L. Raman technologies in cancer diagnostics. *Analyst* **141**(2), 476–503 (2016). 26539569 10.1039/c5an01786f

[14] Diem, M. et al. Applications of infrared and Raman microspectroscopy of cells and tissue in medical diagnostics: present status and future promises. *J. Spectrosc.* **27**(5–6), 463–496 (2012).

[15] Hanlon, E. et al. Prospects for in vivo Raman spectroscopy. *Phys. Med. Biol.* **45**(2), R1 (2000). 10701500 10.1088/0031-9155/45/2/201

[16] Jermyn, M. et al. A review of Raman spectroscopy advances with an emphasis on clinical translation challenges in oncology. *Phys. Med. Biol.* **61**(23), R370 (2016). 27804917 10.1088/0031-9155/61/23/R370

[17] Mahadevan-Jansen, A. & Richards-Kortum, R. R. Raman spectroscopy for the detection of cancers and precancers. *J. Biomed. Opt.* **1**(1), 31–70 (1996). 23014644 10.1117/12.227815

[18] Pence, I. & Mahadevan-Jansen, A. Clinical instrumentation and applications of Raman spectroscopy. *Chem. Soc. Rev.* **45**(7), 1958–1979 (2016). 26999370 10.1039/c5cs00581g PMC4854574

[19] Schie, I. W. & Huser, T. Methods and applications of Raman microspectroscopy to single-cell analysis. *Appl. Spectrosc.* **67**(8), 813–828 (2013). 23876720 10.1366/12-06971

[20] Shipp, D. W., Sinjab, F. & Notingher, I. Raman spectroscopy: Techniques and applications in the life sciences. *Adv. Opt. Photon.* **9**(2), 315–428 (2017).

[21] Stevens, O., Petterson, I. E. I., Day, J. C. & Stone, N. Developing fibre optic Raman probes for applications in clinical spectroscopy. *Chem. Soc. Rev.* **45**(7), 1919–1934 (2016). 26956027 10.1039/c5cs00850f

[22] Tu, Q. & Chang, C. Diagnostic applications of Raman spectroscopy. *Nanomed. Nanotechnol. Biol. Med.* **8**(5), 545–558 (2012). 10.1016/j.nano.2011.09.013 22024196

[23] Wachsmann-Hogiu, S., Weeks, T. & Huser, T. Chemical analysis in vivo and in vitro by Raman spectroscopy—From single cells to humans. *Curr. Opin. Biotechnol.* **20**(1), 63–73 (2009). 19268566 10.1016/j.copbio.2009.02.006 PMC3185305

[24] Han, R., Lin, N., Huang, J. & Ma, X. Diagnostic accuracy of Raman spectroscopy in oral squamous cell carcinoma. *Front. Oncol.* **8**, 12 (2022). 10.3389/fonc.2022.925032 PMC9389172 35992884

[25] Cordero, E., Latka, I., Matthäus, C., Schie, I. & Popp, J. In-vivo Raman spectroscopy: From basics to applications. *J. Biomed. Opt.* **23**(7), 071210 (2018). 10.1117/1.JBO.23.7.071210 29956506

[26] Bergholt, M. S. et al. Combining near-infrared-excited autofluorescence and Raman spectroscopy improves in vivo diagnosis of gastric cancer. *Biosens. Bioelectron.* **26**(10), 4104–4110 (2011). 21550225 10.1016/j.bios.2011.04.005

[27] Bergholt, M. S. et al. In vivo diagnosis of gastric cancer using Raman endoscopy and ant colony optimization techniques. *Int. J. Cancer* **128**(11), 2673–2680 (2011). 20726002 10.1002/ijc.25618

[28] Duraipandian, S. et al. Real-time Raman spectroscopy for in vivo, online gastric cancer diagnosis during clinical endoscopic examination. *J. Biomed. Opt.* **17**(8), 081418 (2012). 23224179 10.1117/1.JBO.17.8.081418

[29] Duraipandian, S. et al. In vivo diagnosis of cervical precancer using Raman spectroscopy and genetic algorithm techniques. *Analyst* **136**(20), 4328–4336 (2011). 21869948 10.1039/c1an15296c

[30] Duraipandian, S., Zheng, W., Ng, J. et al. Integrated fingerprint and high wavenumber confocal Raman spectroscopy for in vivo diagnosis of cervical precancer. In *Advanced Biomedical and Clinical Diagnostic Systems* XI. (SPIE, 2013).

[31] Huang, Z. et al. In vivo early diagnosis of gastric dysplasia using narrow-band image-guided Raman endoscopy. *J. Biomed. Opt.* **15**(3), 037017-037017–5 (2010). 20615046 10.1117/1.3420115

[32] Jermyn, M. et al. Intraoperative brain cancer detection with Raman spectroscopy in humans. *Sci. Transl. Med.* **7**(274), 274ra19 (2015). 25673764 10.1126/scitranslmed.aaa2384

[33] Leblond, F., Jermyn, M., Mok, K. et al., Intraoperative detection of cancerous brain tissue using Raman spectroscopy. SPIE Newsroom (2015).

[34] Lin, K. et al. Rapid fiber-optic Raman spectroscopy for real-time in vivo detection of gastric intestinal metaplasia during clinical gastroscopy. *Cancer Prev. Res.* **9**(6), 476–483 (2016). 10.1158/1940-6207.CAPR-15-0213 27034388

[35] Magee, N. D. et al. Ex vivo diagnosis of lung cancer using a Raman miniprobe. *J. Phys. Chem. B* **113**(23), 8137–8141 (2009). 19453143 10.1021/jp900379w

[36] Teh, S. et al. Near-infrared Raman spectroscopy for early diagnosis and typing of adenocarcinoma in the stomach. *J. Br. Surg.* **97**(4), 550–557 (2010). 10.1002/bjs.6913 20155786

[37] Teh, S. K. et al. Diagnosis of gastric cancer using near-infrared Raman spectroscopy and classification and regression tree techniques. *J. Biomed. Opt.* **13**(3), 034013-034013–8 (2008). 18601558 10.1117/1.2939406

[38] Wang, J. et al. Fiber-optic Raman spectroscopy for in vivo diagnosis of gastric dysplasia. *Faraday Discuss.* **187**, 377–392 (2016). 27048992 10.1039/c5fd00151j

[39] Wang, J. et al. Simultaneous fingerprint and high-wavenumber fiber-optic Raman spectroscopy improves in vivo diagnosis of esophageal squamous cell carcinoma at endoscopy. *Sci. Rep.* **5**(1), 12957 (2015). 26243571 10.1038/srep12957 PMC4525386

[40] Zeng, H., Short, M. A., McWilliams, A. & Lam, S. In vivo Raman spectroscopy for early lung cancer detection. In *Asia Communications and Photonics Conference and Exhibition*. (IEEE, 2010).

[41] Cals, F. L. J. et al. Raman spectroscopic analysis of the molecular composition of oral cavity squamous cell carcinoma and healthy tongue tissue. *Analyst* **143**(17), 4090–4102 (2018). 30083685 10.1039/c7an02106b

[42] Lin, K., Zheng, W., Lim, C. M. & Huang, Z. Real-time in vivo diagnosis of laryngeal carcinoma with rapid fiber-optic Raman spectroscopy. *Biomed. Opt. Express* **7**(9), 3705–3715 (2016). 27699131 10.1364/BOE.7.003705 PMC5030043

[43] Cals, F. L. et al. Development and validation of Raman spectroscopic classification models to discriminate tongue squamous cell carcinoma from non-tumorous tissue. *Oral Oncol.* **60**, 41–47 (2016). 27531871 10.1016/j.oraloncology.2016.06.012

[44] Cals, F. L. et al. Investigation of the potential of Raman spectroscopy for oral cancer detection in surgical margins. *Lab. Invest.* **95**(10), 1186–1196 (2015). 26237270 10.1038/labinvest.2015.85

[45] Connolly, J. M. et al. Non-invasive and label-free detection of oral squamous cell carcinoma using saliva surface-enhanced Raman spectroscopy and multivariate analysis. *Nanomed. Nanotechnol. Biol. Med.* **12**(6), 1593–1601 (2016). 10.1016/j.nano.2016.02.021 27015768

[46] Ding, J. et al. Diverse spectral band-based deep residual network for tongue squamous cell carcinoma classification using fiber optic Raman spectroscopy. *Photodiagn. Photodyn. Ther.* **32**, 102048 (2020). 10.1016/j.pdpdt.2020.102048 33017657

[47] Jeng, M.-J. et al. Raman spectroscopy analysis for optical diagnosis of oral cancer detection. *J. Clin. Med.* **8**(9), 1313 (2019). 31461884 10.3390/jcm8091313 PMC6780219

[48] Jeng, M.-J. et al. Novel quantitative analysis using optical imaging (Velscope) and spectroscopy (Raman) techniques for oral cancer detection. *Cancers* **12**(11), 3364 (2020). 33202869 10.3390/cancers12113364 PMC7696965

[49] Knipfer, C. et al. Raman difference spectroscopy: a non-invasive method for identification of oral squamous cell carcinoma. *Biomed. Opt. Express* **5**(9), 3252–3265 (2014). 25401036 10.1364/BOE.5.003252 PMC4230857

[50] Krishna, H., Majumder, S. K., Chaturvedi, P., Sidramesh, M. & Gupta, P. K. In vivo Raman spectroscopy for detection of oral neoplasia: A pilot clinical study. *J. Biophoton.* **7**(9), 690–702 (2014). 10.1002/jbio.201300030 23821433

[51] Malik, A. et al. In vivo Raman spectroscopy–assisted early identification of potential second primary/recurrences in oral cancers: an exploratory study. *Head Neck* **39**(11), 2216–2223 (2017). 28736959 10.1002/hed.24884

[52] Matthies, L. et al. Optical diagnosis of oral cavity lesions by label-free Raman spectroscopy. *Biomed. Opt. Express* **12**(2), 836–851 (2021). 33680545 10.1364/BOE.409456 PMC7901324

[53] Sahu, A. K. et al. Oral cancer screening: Serum Raman spectroscopic approach. *J. Biomed. Opt.* **20**(11), 115006–115006 (2015). 26580700 10.1117/1.JBO.20.11.115006

[54] Sharma, M., Jeng, M.-J., Young, C.-K., Huang, S.-F. & Chang, L.-B. Developing an algorithm for discriminating oral cancerous and normal tissues using Raman spectroscopy. *J. Personal. Med.* **11**(11), 1165 (2021). 10.3390/jpm11111165 PMC8623962 34834517

[55] Tan, Y. et al. Surface-enhanced Raman spectroscopy of blood serum based on gold nanoparticles for the diagnosis of the oral squamous cell carcinoma. *Lipids Health Dis.* **16**, 1–9 (2017). 28388900 10.1186/s12944-017-0465-y PMC5384146

[56] Eilers, P. H. C. A perfect smoother. *Anal. Chem.* **75**(14), 3631–3636 (2003). 14570219 10.1021/ac034173t

[57] Desroches, J. et al. Characterization of a Raman spectroscopy probe system for intraoperative brain tissue classification. *Biomed. Opt. Express* **6**(7), 2380–2397 (2015). 26203368 10.1364/BOE.6.002380 PMC4505696

[58] Short, M. A., Wang, W., Tai, I. T. & Zeng, H. Development and in vivo testing of a high frequency endoscopic Raman spectroscopy system for potential applications in the detection of early colonic neoplasia. *J. Biophoton.* **9**(1–2), 44–48 (2016). 10.1002/jbio.201500205 26587679

[59] Lin, D. et al. Autofluorescence and white light imaging-guided endoscopic Raman and diffuse reflectance spectroscopy for in vivo nasopharyngeal cancer detection. *J. Biophoton.* **11**(4), e201700251 (2018). 10.1002/jbio.201700251 29239125

[60] Lin, K., Cheng, D. L. P. & Huang, Z. Optical diagnosis of laryngeal cancer using high wavenumber Raman spectroscopy. *Biosens. Bioelectron.* **35**(1), 213–217 (2012). 22465448 10.1016/j.bios.2012.02.050

[61] Lin, K., Zheng, W., Lim, C. M. & Huang, Z. Real-time in vivo diagnosis of nasopharyngeal carcinoma using rapid fiber-optic Raman spectroscopy. *Theranostics* **7**(14), 3517–3526 (2017). 28912892 10.7150/thno.16359 PMC5596440

[62] Ming, L. C. et al. Real time near-infrared Raman spectroscopy for the diagnosis of nasopharyngeal cancer. *Oncotarget* **8**(30), 64 (2017). 28533478 10.18632/oncotarget.17703 PMC5564780

[63] Žuvela, P. et al. Fiber-optic Raman spectroscopy with nature-inspired genetic algorithms enhances real-time in vivo detection and diagnosis of nasopharyngeal carcinoma. *Anal. Chem.* **91**(13), 8101–8108 (2019). 31135136 10.1021/acs.analchem.9b00173

[64] Chen, J. W. et al. Detection of adenosine using surface-enhanced Raman scattering based on structure-switching signaling aptamer. *Biosens. Bioelectron.* **24**(1), 66–71 (2008). 18436440 10.1016/j.bios.2008.03.013

[65] De Gelder, J., De Gussem, K., Vandenabeele, P. & Moens, L. Reference database of Raman spectra of biological molecules. *J. Raman Spectrosc.* **38**(9), 1133–1147 (2007).

[66] De Veld, D. et al. Autofluorescence and Raman microspectroscopy of tissue sections of oral lesions. *Lasers Med. Sci.* **19**, 203–209 (2005). 15772873 10.1007/s10103-004-0325-7

[67] Deshmukh, A., Singh, S., Chaturvedi, P. & Krishna, C. M. Raman spectroscopy of normal oral buccal mucosa tissues: study on intact and incised biopsies. *J. Biomed. Opt.* **16**(12), 127004–127004-10 (2011). 22191934 10.1117/1.3659680

[68] Guo, J., Cai, W., Du, B., Qian, M. & Sun, Z. Raman spectroscopic investigation on the interaction of malignanthepatocytes with doxorubicin. *Biophys. Chem.* **140**(1–3), 57–61 (2009). 19070416 10.1016/j.bpc.2008.11.005

[69] Hanahan, D. & Weinberg, R. A. The hallmarks of cancer. *Cell* **100**(1), 57–70 (2000). 10647931 10.1016/s0092-8674(00)81683-9

[70] Li, Y. et al. Research on the Raman spectral character and diagnostic value of squamous cell carcinoma of oral mucosa. *J. Raman Spectrosc.* **41**(2), 142–147 (2010). 20337078

[71] Lo, W. L. et al. Raman spectroscopy monitoring of the cellular activities of a tissue-engineered ex vivo produced oral mucosal equivalent. *J. Raman Spectrosc.* **42**(2), 174–178 (2011).

[72] Schweitzer-Stenner, R. Advances in vibrational spectroscopy as a sensitive probe of peptide and protein structure: A critical review. *Vib. Spectrosc.* **42**(1), 98–117 (2006).

[73] Uzunbajakava, N. et al. Nonresonant confocal Raman imaging of DNA and protein distribution in apoptotic cells. *Biophys. J.* **84**(6), 3968–3981 (2003). 12770902 10.1016/S0006-3495(03)75124-8 PMC1302978

[74] Valdés, R., Stefanov, S., Chiussi, S., López-Alvarez, M. & González, P. Pilot research on the evaluation and detection of head and neck squamous cell carcinoma by Raman spectroscopy. *J. Raman Spectrosc.* **45**(7), 550–557 (2014).

[75] Abbasi, H. et al. Development of a near-infrared Raman spectroscopy setup compatible with fluorescence-guided surgery. *Analyst* **148**(12), 2676–2682 (2023). 37077171 10.1039/d3an00077j

[76] Bali, A. et al. Endoscopic in vivo hyperspectral imaging for head and neck tumor surgeries using a medically approved CE-certified camera with rapid visualization during surgery. *Cancers (Basel).* **16**(22), 3785 (2024). 39594741 10.3390/cancers16223785 PMC11592278

[77] van Lanschot, C. G. F. et al. Relocation of inadequate resection margins in the wound bed during oral cavity oncological surgery: A feasibility study. *Head Neck* **41**(7), 2159–2166. 10.1002/hed.25690 (2019). 30706624 10.1002/hed.25690 PMC6618026

